# Modulation of Adaptive Immunity and Viral Infections by Ion Channels

**DOI:** 10.3389/fphys.2021.736681

**Published:** 2021-10-08

**Authors:** Karen Bohmwald, Nicolás M. S. Gálvez, Catalina A. Andrade, Valentina P. Mora, José T. Muñoz, Pablo A. González, Claudia A. Riedel, Alexis M. Kalergis

**Affiliations:** ^1^Departamento de Genética Molecular y Microbiología, Facultad de Ciencias Biológicas, Millennium Institute on Immunology and Immunotherapy, Pontificia Universidad Católica de Chile, Santiago, Chile; ^2^Departamento de Ciencias Biológicas, Facultad de Ciencias de la Vida, Millennium Institute on Immunology and Immunotherapy, Universidad Andres Bello, Santiago, Chile; ^3^Departamento de Endocrinología, Facultad de Medicina, Pontificia Universidad Católica de Chile, Santiago, Chile

**Keywords:** Ca^2+^, Ca^2+^ channels, T cells, immune response, viral infection

## Abstract

Most cellular functions require of ion homeostasis and ion movement. Among others, ion channels play a crucial role in controlling the homeostasis of anions and cations concentration between the extracellular and intracellular compartments. Calcium (Ca^2+^) is one of the most relevant ions involved in regulating critical functions of immune cells, allowing the appropriate development of immune cell responses against pathogens and tumor cells. Due to the importance of Ca^2+^ in inducing the immune response, some viruses have evolved mechanisms to modulate intracellular Ca^2+^ concentrations and the mobilization of this cation through Ca^2+^ channels to increase their infectivity and to evade the immune system using different mechanisms. For instance, some viral infections require the influx of Ca^2+^ through ionic channels as a first step to enter the cell, as well as their replication and budding. Moreover, through the expression of viral proteins on the surface of infected cells, Ca^2+^ channels function can be altered, enhancing the pathogen evasion of the adaptive immune response. In this article, we review those ion channels and ion transporters that are essential for the function of immune cells. Specifically, cation channels and Ca^2+^ channels in the context of viral infections and their contribution to the modulation of adaptive immune responses.

## Introduction

**FIGURE 1:**
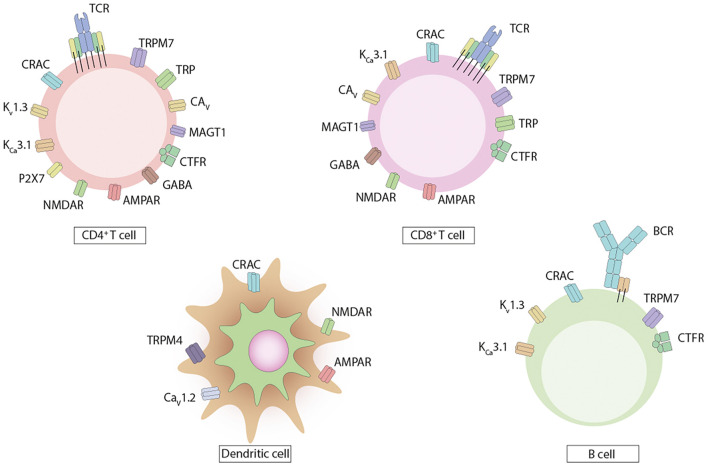
Ion channels and transporters expressed by dendritic cells and lymphocytes. The immune cells express on their surface various types of ion channels and transporters. CD4^+^ and CD8^+^ T cells express channels for Ca^2+^, Mg^2+^, K^+^, and Cl^–^, with fewer channels reported on CD8^+^ T cells. B cells express channels for Ca^2+^, Mg^2+^, and K^+^. Dendritic cells express Ca^2+^ channels.

Ion homeostasis must be strictly modulated in cells of the immune system since these charged atoms or molecules play critical roles in several different physiological aspects (Feske et al., 2015; Rubaiy, 2017). Cations are mainly represented by calcium (Ca^2+^), magnesium (Mg^2+^), zinc (Zn^2+^), potassium (K^+^), and sodium (Na^+^), and anions by chloride (Cl^–^), phosphate (HPO_4_^2–^), and bicarbonate (HCO3^–^) (Feske et al., 2015; Rubaiy, 2017; Vaeth and Feske, 2018). Na^+^, K^+^, and Cl^–^ usually maintain electrical gradients, and these cations contribute to the survival of the cell along with the movement of organelles, while Cl^–^ concentration plays a role in the movement and the acidification process of organelles (Feske et al., 2012, 2015; Chakraborty et al., 2017; Murray et al., 2017; Rubaiy, 2017; Vaeth and Feske, 2018). Therefore, the mobilization of these charged atoms or molecules across the hydrophobic plasma membrane alters the electrochemical gradient in a localized fashion, inducing conformational changes that will influence the function of membrane proteins on those specific portions of the membrane (Feske et al., 2012, 2015; Chaigne-Delalande and Lenardo, 2014; Rubaiy, 2017; Vaeth and Feske, 2018). Given the relevance of ion channels and transporters in cellular functions, it is expected for immune cells, such as dendritic cells (DCs) and lymphocytes, to express several of these proteins for a specialized regulation of ion transport (Figure 1; Feske et al., 2012, 2015; Rubaiy, 2017; Vaeth and Feske, 2018). Overall, these channels are plasma membrane integral proteins that allow ions to transit from one side of the membrane to the other (Di Resta and Becchetti, 2010; Kim, 2014). Usually, cellular channels can be found in an open, resting closed, or inactivated closed state, in line with the physiological conditions and needs of the cell (Di Resta and Becchetti, 2010; Kim, 2014).

**FIGURE 2:**
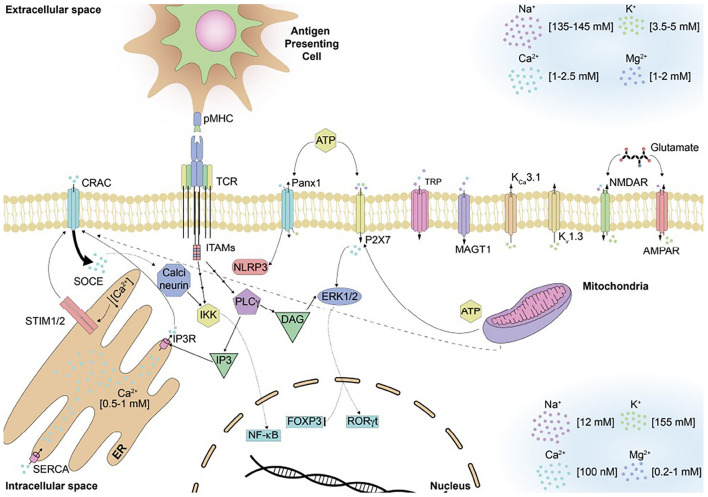
Ion channels and signaling pathways described for T cells. Several channels responsible of regulating the ion homeostasis can be found on the surface of T cells. These channels maintain the different concentrations of ion in the extracellular and intracellular space and are permeable to specific ions. T cell receptor engagement with the pMHC molecule found on antigen presenting cells will induce a signaling cascade that will result on the influx of Ca^2+^ and eventual store-operated Ca^2+^ entry inside of T cells. All these events will result in the activation of different transcription factor that will activate the effector function of T cells.

Although currently there is not a thorough characterization regarding the modulation of ion channels and transporters in immune cells during infectious diseases, some reports have provided relevant information to understand better the contribution of these molecules during an infection (Cahalan and Chandy, 1997; Panyi et al., 2004; Feske et al., 2012, 2015; RamaKrishnan and Sankaranarayanan, 2016; Vaeth and Feske, 2018). It has been described that both, ion channels and transporters can regulate cation transport and modulate the immune response (Zweifach and Lewis, 1993; Lewis, 2001; Hogan et al., 2010; Feske et al., 2012). The most relevant ion channels and transporters include the Ca^2+^ release-activated Ca^2+^ (CRAC) channels, voltage-gated such as K^+^ channel (K_v_)1.3 and Ca^2+^-activated K^+^ channel (K_Ca_)3.1, transient receptor potential (TRP) channels, P2X receptors family and pannexin hemichannels, voltage-gated Ca^2+^, Na^+^, and H^+^ channels, and Mg^2+^ and Zn^2+^ channels, among others (Zweifach and Lewis, 1993; Lewis, 2001; Schenk et al., 2008; Franchi et al., 2009; Yip et al., 2009; Hogan et al., 2010; Woehrle et al., 2010; Junger, 2011; Feske et al., 2012; Prakriya and Lewis, 2015; Figure 2). One of the ion channels that regulates anion transport, and the immune response is the cystic fibrosis transmembrane conductance regulator (CFTR) and gamma-aminobutyric acid (GABA) receptors. In general, Ca^2+^ channel signaling starts with the influx of Ca^2+^ from the extracellular space, which triggers the activation of phospholipase Cβ (PLCβ) and the subsequent cleavage of phosphatidylinositol 4,5-bisphosphate (PIP_2_) into inositol (1,4,5) trisphosphate (IP_3_) and diacylglycerol (DAG) by PLCγ. After this, the binding of IP_3_ with to the IP_3_-receptor (IP_3_R) on the endoplasmic reticulum (ER) allows the diffusion of Ca^2+^ into the cytoplasm (Clapham, 2007). The following sections will discuss the contribution of ion channels and transporters to the interface between antigen-presenting cells, such as DCs and T cells, an interaction commonly known as immunological synapse. Further, the modulation of these channels during adaptive immune responses, diseases, channelopathies, and viral infections will be discussed.

## Ion Channels Expressed by T and B Cells

T cells and B cells are the main players of the adaptive immune response and express various ion channels and transporters on their surface (Feske et al., 2012; Netea et al., 2019). These channels can regulate the concentration of different ions inside these cells and modulate their functions (Feske et al., 2012). This section will discuss the ion channels and transporters expressed by these lymphocytes.

### Ion Channels Expressed by T Cells

T cells play an essential role at connecting the humoral and cellular arms of adaptive immunity, which is critical to properly response to infections by various pathogens (Jasenosky et al., 2015; Lee et al., 2020). T cells can be classified into two major types: CD4^+^ T cells that orchestrate the immune response against a specific pathogen, and CD8^+^ T cells that have cytotoxic properties and can induce the apoptosis of cells infected by a pathogen (Whitacre et al., 2012; Jasenosky et al., 2015). In general, these cells can be recognized for the T cell receptor found on their surface (Whitacre et al., 2012). Additionally, T cells express several ion channels and transporters that modulate the activation of these cells through the influx of Ca^2+^, Mg^2+^ and Zn^2+^, or the efflux of K^+^ and Cl^–^ (Table 1; Feske, 2013; Feske et al., 2015).

**TABLE 1:** Role of ion channels expressed by lymphocytes.

Ion to be modulated	Channels	Role in the lymphocytes functioning		References
		**T cells**	**B cells**	
Ca^2+^	CRAC	Increment of [Ca^2+^]_i_ upon T cell receptor engagement. Participates in the secretion of cytokines.	Increment of [Ca^2+^]_i_ upon B cell receptor engagement Modulates the proliferation of B cells. Modulates the secretion of antibodies.	Lewis, 2001; Feske et al., 2012; Prakriya and Lewis, 2015; Trebak and Kinet, 2019
	P2X	Amplifies gene expression, T cell effector functions, and T cell receptor signaling.	Not expressed by this cell type	Schenk et al., 2008; Woehrle et al., 2010; Junger, 2011
	TRP	Enhances T cell receptor stimulation. Regulates differentiation of T cell into Th1 or Th2 profile. Induces proliferation.	Not expressed by this cell type	Weber et al., 2010; Acharya et al., 2021
	Ca_v_	Secretion of IL-2. Differentiation of T cells into Th2 profiles. Homeostasis of naïve T cells.	Not expressed by this cell type	Cabral et al., 2010; Omilusik et al., 2011; Fenninger and Jefferies, 2019
Mg^2+^	TRPM7	Promotes T cell development and maturation.	Might be essential for B cell survival.	Jin et al., 2008; Feske et al., 2012; Brandao et al., 2013
	MagT1	Induces the activation of T cells.	Does not induce the activation of B cells.	Goytain and Quamme, 2005
K^+^	K_v_	Promotes the central and effector memory phenotypes of T cells.	Promotes the secretion of autoantibodies. Induces proliferation of class-switching memory B cells	Chiang et al., 2017
	K_Ca_		Unknown role	
Zn^2+^	ZIP	Unknown role	Unknown role	Feske et al., 2012
Cl^–^	CAGA	Inhibition of T cell function and proliferation.	Not expressed by this cell type	Feske et al., 2012
	CFTR	Regulates differentiation profile of T cells.	Activation and proliferation of B cells.	Polverino et al., 2019

#### Calcium Channels

CRAC channels contribute significantly to modulating immune cells, particularly the T cells, as these channels are mainly responsible for Ca^2+^ influx, promoting an increase in the concentration of this ion that can be maintained for hours after the initial opening of these channels (Lewis, 2001; Joseph et al., 2014). Ca^2+^ levels modulate the activation, proliferation, and cytokine secretion by T lymphocytes, among other relevant processes (reviewed later; Lewis, 2001). CRAC channels are located on the membrane of T cells and are composed of three proteins, ORAI1, ORAI2, and ORAI3 (Vaeth et al., 2020). CRAC channels are also the main Ca^2+^ channels activated on antigen-presenting cells upon antigen recognition, promoting DCs maturation in a Ca^2+^-controlled fashion (Prakriya and Lewis, 2015). Usually, Ca^2+^ concentration in the cytoplasm of resting lymphocytes is close to 50 to 100 nM (Figure 2). Upon T cell receptor or B cell receptor engagement, Ca^2+^ concentration in the cytoplasm can reach up to 1 μM (Lewis, 2001; Feske et al., 2012). The mechanisms underlying Ca^2+^ increase after T cell receptor and B cell receptor engagement involves the activation of PLCγ1 or PLCγ2 (Zweifach and Lewis, 1993; Hogan et al., 2003). The production of IP_3_ by PLCγ leads to the release of Ca^2+^ from the ER that will stimulate Ca^2+^ influx across the plasma membrane through the store-operated Ca^2+^ entry (Zweifach and Lewis, 1993; Hogan et al., 2010). CRAC channels are responsible for the store-operated Ca^2+^ entry, and they are activated upon binding to the stromal interaction molecule 1 (STIM1) and ORAI 1–3 proteins (Hogan et al., 2010). Notably, the activation of CRAC channels occurs after the ORAI proteins bind to STIM proteins at the ER-plasma membrane junction (ORAI binding site to STIM is located at the carboxyl terminus of the former; Feske et al., 2012; Joseph et al., 2014). Upon binding, STIM proteins sense Ca^2+^ depletion inside the ER, oligomerizing and redistributing toward these junctions where they communicate this Ca^2+^ decrease to CRAC channels (Hogan et al., 2003; Feske et al., 2012). The cytoplasmic domain of STIM1 interacts with the N and C termini in ORAI1, and CRAC channels are activated, leading to the store-operated Ca^2+^ entry (Feske et al., 2012). STIM2, another isoform of STIM proteins, is expressed in lymphocytes and can interact with ORAI1, although with slower kinetics than STIM1 (Feske et al., 2012). Activation of CRAC channels and the store-operated Ca^2+^ entry induction is key for cytokine production because CD4^+^ and CD8^+^ T cells from STIM1-deficient mice and ORAI1 cannot secrete cytokines, such as IL-2, IL-4, IL-17, TNF, and IFN-γ (Trebak and Kinet, 2019). In T cells, mitochondria work alongside CRAC channels and interact with them to maintain Ca^2+^ influx and regulate homeostasis (Joseph et al., 2014). After T cell receptor engagement and immunological synapsis formation, mitochondria moves closes to CRAC channels in response to the massive transportation of Ca^2+^, thus prolonging CRAC activity (Joseph et al., 2014). Figure 2 describes these events on T cells upon T cell receptor engagement by cognate peptide-MHC (pMHC) complexes on the antigen-presenting cells surface.

The modulation of the Ca^2+^ concentrations inside T cells is also mediated by plasma membrane Ca^2+^ ATPase pumps (PMCAs). During immunological synapsis, PMCAs are in close contact with CRAC channels, which modulates the activity of the ATPase pump. However, PMCAs efflux of Ca^+2^ from the T cell is reduced, to allow mitochondrial control of Ca^+2^ flux (Quintana and Hoth, 2012). PMCAs also promote the activation the sarco-endoplasmic reticulum Ca^2+^ ATPase (SERCA), which removes cytosolic Ca^2+^ into the ER and the mitochondria (Hogan et al., 2003; Korthals et al., 2017). In addition, ryanodine receptor channels also increase the efflux of Ca^2+^ from the ER to the cytoplasm (Fenninger and Jefferies, 2019). These channels are activated by the release of cyclic adenosine diphosphate (ADP) ribose (cADPR) induced upon the store-operated Ca^2+^ entry (Fenninger and Jefferies, 2019). PMCAs are activated after Ca^2+^-loaded calmodulin binds to their carboxyl terminus (Joseph et al., 2014; Fenninger and Jefferies, 2019). Although PMCAs have a high affinity for Ca^2+^, they also have low permeability for this cation, which is why they modulate Ca^2+^ concentration at basal levels (Joseph et al., 2014).

The P2X purinoreceptor ion-channel family (and specifically P2X1, P2X2, and P2X7) consists of another group of channels related to immunity and myeloid and lymphoid cell modulation (Junger, 2011). These channels require extracellular ATP for activation and are non-selective for cations, such as Ca^2+^ and Na^+^ (Junger, 2011). P2X7 receptors mainly control complex pathways that modulate Ca^2+^ and Na^+^ influx and K^+^ efflux and are primarily expressed by T lymphocytes and innate immune cells (Yip et al., 2009; Junger, 2011). Activation of P2X7 by ions such as K^+^, Na^+^, Ca^2+^, and Cl^–^ results in significant stimulation of the NLRP3 inflammasome in several innate cells, such as in macrophages and monocytes (Yip et al., 2009; Junger, 2011). Furthermore, NLRP3 oligomerization and inflammasome activation will result in Caspase 1-induced cleavage of pro-IL-1β and pro-IL-18 into their mature forms, IL-1β and IL-18 (Franchi et al., 2009). These cytokines will activate additional signaling pathways to induce inflammation and immune cell function (Franchi et al., 2009). Remarkably, Pannexin 1 hemichannels (Panx1) has been reported to co-localize with P2X7 at the immunological synapsis, (Woehrle et al., 2010). Because Panx1 can release ATP into the extracellular space, it has been suggested that T cells can modulate P2X7 signaling (Woehrle et al., 2010). This modulation can work in an autocrine fashion to amplify gene expression, T cell effector functions, and T cell receptor signals upon pMHC engagement (Schenk et al., 2008; Woehrle et al., 2010; Junger, 2011; Figure 2). Activation of the inflammasome and Caspase-1 is also linked to programmed cell death (Martinon et al., 2002; Shi et al., 2017). Gasdermin D is a target of Caspase-1, -4, -5, and -11 and has been identified as a key component of the molecular mechanisms of pyroptosis (Lamkanfi et al., 2008; Shi et al., 2015). Pyroptosis is a cellular phenomenon leading to programmed cell death that increases the presence of inflammatory molecules in the extracellular space, such as IL-1β (Shi et al., 2017), which differs from non-inflammatory cell death triggered by apoptosis (Jorgensen and Miao, 2015). The inflammatory features of pyroptosis include the activation of PRRs and an increased transcription of pro-inflammatory cytokines, which classifies this process as an innate immunity-related mechanism (Jorgensen and Miao, 2015). Gasdermin D is usually found in an inactivated state and, upon cleavage by the aforementioned Caspases, can act as a pore-forming protein by disrupting the plasma membrane, cell swelling, and lysis, in a pyroptotic fashion (Ding et al., 2016; Liu et al., 2016). Remarkably, increased cytosolic Ca^2+^ leads to increased translocation of GSDMD into the membrane and enhanced LPS-induced pyroptosis, in a PLCγ1-dependent manner (Liu et al., 2020). Therefore, Ca^2+^ contributes to the modulation of programmed cell death.

Transient receptor potential channels were initially described in *Drosophila melanogaster* mutants and constitute a superfamily including 6 subfamilies in humans: TRP canonical (TRCP), TRP vanilloid (TRPV), TRP melastatin (TRPM), TRP ankyrin (TRPA), TRP mulcolipin, and TRP polycystic (Minke and Cook, 2002; Wenning et al., 2011; Feske et al., 2012; Samanta et al., 2018). The most common TRP receptors expressed by T cells are TRPC and TRPM (Parenti et al., 2016; Froghi et al., 2021). TRPC channels are thought to be responsible for Ca^2+^ influx into T cells and are usually associated with PLCγ signaling (Feske et al., 2012). Further studies are required to elucidate if these channels contribute to the store-operated Ca^2+^ entry and T cell activation (Minke and Cook, 2002; Feske et al., 2012). TRP cation channel subfamily M member 2 (TRPM2) channels have been shown to regulate Ca^2+^ in a non-selective fashion in several immune cells (Yamamoto et al., 2010). These channels are regulated by several intracellular factors, mostly related to ADP, such as cADP and cADPR (Yamamoto et al., 2010; Huang et al., 2018). Other molecules, such as hydrogen peroxide, may modulate the permeability and activity of TRPM2 (Yamamoto et al., 2010; Sumoza-Toledo and Penner, 2011; Wenning et al., 2011). Different reports have shown that these channels can enhance T cell receptor stimulation in T cells and inhibit reactive oxygen species production in phagocytic cells (Di et al., 2011; Chung et al., 2015; Syed Mortadza et al., 2015; Morad et al., 2021). Furthermore, this channel can promote the secretion of cytokines such as IL-2, IL-17, and IFN-γ (Melzer et al., 2012). TRPM4, the most studied TRP channels in immune cells, are highly permeable to Na^+^ and K^+^ and partially to Ca^2+^ (Vennekens and Nilius, 2007). The influx of Ca^2+^ will induce the activation of TRPM4, which will negatively modulate store-operated Ca^2+^ entry and promote Na^+^ influx, membrane depolarization, and reduction of Ca^2+^ influx due to stabilizing electrical charges (Vennekens and Nilius, 2007; Feske et al., 2012; Bose et al., 2015). The modulation of Ca^2+^ levels regulates the Th1 and Th2 differentiation of T cells (Weber et al., 2010). Lastly, TRPM8 has been associated to T cell activation, increasing CD25 and CD69 expression and TNF-α secretion (Acharya et al., 2021). Although TRPM8 might not be necessary for T cell activation, inhibition of this channel impairs T cell proliferation (Acharya et al., 2021).

Ca_v_ channels induce specific Ca^2+^ influx in excitable cells and are divided into three subfamilies, Ca_v_1, Ca_v_2, and Ca_v_3. T cells express only the Ca_v_1 first subfamily (Feske et al., 2012, 2015), which comprises Ca_v_1.1, Ca_v_1.2, Ca_v_1.3, Ca_v_1.4, and the regulatory subunits β3 and β4 (Feske et al., 2012, 2015; Badou et al., 2013). Treatment of Jurkat T cells with a Ca_v_ channel antagonists reduces IL-2 production (Fenninger and Jefferies, 2019), suggesting the involvement of this channel in the secretion of this cytokine. The upregulation of Ca_v_ 1.2 and Ca_v_ 1.3 channels and the differentiation of T cells toward a Th2 profile are also associated (Cabral et al., 2010). Ca_v_1.2 activity is also regulated via STIM, as upon inhibition of Ca_v_ 1.2, the interaction between STIM and ORAI occurs (Badou et al., 2013). The influx of Ca^2+^ generated by Ca_v_ 1.4 channels modulates the homeostasis of naïve T cells and is important for the generation of T cell responses (Omilusik et al., 2011).

#### Magnesium Transporters and Channels

Magnesium is another secondary messenger important for modulating the function of T cells, which has been associated with promoting the correct function and development of these cells (Feske et al., 2012). Magnesium transporter protein 1 (MAGT1, an Mg^2+^ transporter) is a highly selective Mg^2+^ channel that is not permeable to other divalent cations such as Ca^2+^, Zn^2+^, and Ni^2+^ (Figure 2; Goytain and Quamme, 2005; Ravell et al., 2020) and is involved in T cell development and function (Goytain and Quamme, 2005). Notably, MAGT1 will induce PLCγ1 activation and then store-operated Ca^2+^ entry, upon Mg^2+^ influx and T cell receptor stimulation (Ravell et al., 2020). T cells lacking MAGT1 channels exhibit an impaired capacity to be activated upon T cell receptor engagement and show a decreased Ca^2+^ influxes (Li et al., 2011; Brandao et al., 2013).

TRP cation channel subfamily M member 7 is a non-selective Mg^2+^ channel expressed by T cells, permeable also to Ca^2+^ (Bates-Withers et al., 2011; Antunes et al., 2016; Vaeth and Feske, 2018). TRPM7 channels regulate Mg^2+^ concentration and cell homeostasis in the entire organism (Bates-Withers et al., 2011; Antunes et al., 2016; Vaeth and Feske, 2018) and are fundamental for T cell development and maturation (Jin et al., 2008; Feske et al., 2012). The absence of this channel impairs the development of T cells, resulting in CD4^–^ and CD8^–^ T cells (Schmitz et al., 2003; Feske et al., 2012). However, it seems that the lack of TRPM7 does not modify the intracellular concentration of Mg^2+^ ([Mg^2+^]_i_) in T cells, but rather the primary function of this channel might be related to Ca^2+^ influx (Feske et al., 2012). The translocation of Ca^2+^ or Mg^2+^ into target cell leads to the phosphorylation of molecules involved in the signaling of tyrosine kinases-based receptors, promoting cell differentiation, growth and activation (Zou et al., 2019).

#### Potassium Channels

The K_v_1.3 and K_Ca_3.1 channels modulate K^+^ homeostasis (Chiang et al., 2017). These channels must control the efflux of K^+^, which leads to hyperpolarization of the plasma membrane (Lam and Wulff, 2011; Chiang et al., 2017). These channels inhibit membrane depolarization upon the influx of other cations, such as Mg^2+^ and Ca^2+^ (Lam and Wulff, 2011). The topology of K_v_1.3 and K_Ca_3.1 is very similar, as they are homotetramers with each subunit exhibiting six transmembrane segments (Di Lucente et al., 2018; Ji et al., 2018). However, these channels differ in the mechanism of activation (Wulff et al., 2009). While K_v_1.3 is activated upon membrane depolarization leading to K^+^ efflux, K_Ca_3.1 activation requires the release of Ca^2+^ and calmodulin. This process leads to repolarization of the plasma membrane upon K^+^ efflux and continuous influx of Ca^2+^ (Wulff et al., 2009). Interestingly, these channels are relevant for establishing central and effector memory phenotypes in T cells (Chiang et al., 2017).

#### Additional Cationic Channels

*N*-methyl-D-aspartate receptors (NMDARs) and α-amino-3-hydroxy-5-methyl-4-isoxazole propionic acid receptors (AMPARs) are ionotropic glutamate receptors that deserve mention, as they are considered receptors linked to ion channels and are relevant during glutamatergic transmission in the central nervous system (CNS; Traynelis et al., 2010). Glutamate is considered one of the primary excitatory neurotransmitters of the CNS in mammals, and it can interact with ionotropic and metabotropic receptors (Traynelis et al., 2010). NMDARs and AMPARs are, as many of the channels mentioned so far, non-selective cations channels that are mostly permeable to K^+^ and Na^+^, and to some extent to Ca^2+^ (Traynelis et al., 2010). These receptors will produce excitatory post-synaptic responses upon activation (Fourgeaud et al., 2010; Traynelis et al., 2010; Kahlfuß et al., 2014). These receptors can also be found in several isoforms, depending on the subunit composition (Fourgeaud et al., 2010; Traynelis et al., 2010; Kahlfuß et al., 2014). Recent reports have described that immune cells, such as DCs are sensitive to and can release glutamate (Fourgeaud et al., 2010; Kahlfuß et al., 2014). Further, AMPAR and NMDAR are expressed by some immune cells, suggesting that these receptors can modulate the development, activation, or effector functions of these immune cells (Fourgeaud et al., 2010; Kahlfuß et al., 2014). However, more exhaustive studies are required to better define the contribution of these channels and the associated signaling pathways to the function of T cells and other immune cells.

Additional ion channels can be found on the surface of T cells, such as Zn^2+^ channels -i.e., ZIP3, ZIP6, ZIP8, ZIP10, ZIP14-, but the contribution of these channels to the function of T cells has not been defined (Feske et al., 2012).

#### Anionic Channels

CFTR is one of the anionic channels expressed by T cells (Feske et al., 2012). CFTR promotes the efflux of Cl^–^ from the intracellular to the extracellular and its absence on CD4^+^ T cells induces a Th2 polarization leading to an increase in the secretion of IL-4 (Feske et al., 2012; Polverino et al., 2019). Another Cl^–^ channel in T cells is GABA receptors, and the activation of this receptor has been described to inhibit the function and proliferation of these cells (Feske et al., 2012). Additionally, the treatment with GABA has prevented the secretion of IL-2 and IFN-γ on CD8^+^ T cells (Feske et al., 2012). Finally, the LRRC8-A channel has been identified on T cells and shown to be important for T cell development, function and survival (Kumar et al., 2014; Serra et al., 2021).

### Ion Channels Expressed by B Cells

B cells can act as professional antigen-presenting cells that can prime T cells and can also differentiate into plasma cells to produce antibodies, which bind to specific antigens of pathogens, promoting their clearance (Whitacre et al., 2012; Häusser-Kinzel and Weber, 2019). B cells express the B cell receptor on their surface (Whitacre et al., 2012) and several ion channels and transporters that modulate their activation and function, through the influx of Ca^2+^, Mg^2+^, and Zn^2+^, or the efflux of K^+^ and Cl^–^ (Scharenberg et al., 2007; Feske et al., 2015; Table 1).

#### Calcium Channels

Ca^2 +^ ions act as a second messenger in B cells and are required for proliferation and antibody secretion (Vig and Kinet, 2009; Feske et al., 2012; Trebak and Kinet, 2019). Ca^2+^ influx in B cells is controlled by CRAC channels, which are activated upon antigen recognition (Feske et al., 2012, 2015). Therefore, CRAC channels contribute to the establishment of immunity against infections, modulating B cell proliferation and antibody secretion by these cells. The lack of ORAI1 in B cells can lead to immunodeficiency and also to autoimmunity (Feske et al., 2012). However, the absence of ORAI1 does not affect B cell development (Mahtani and Treanor, 2019). Autoimmune disorders described in the absence of ORAI1 are associated with inflammation of the CNS (Gauthier et al., 2006; Feske et al., 2012). For instance, multiple sclerosis has been related to reduced production of IL-10 by B cells that have mutations associated with CRAC channels (Gauthier et al., 2006; Feske et al., 2012).

#### Magnesium Channels

Mg^2+^ is a secondary messenger essential in B cells because the lack of this cation prevents the proliferation of these cells (Feske et al., 2012). TRPM7 is the Mg^2+^ channel described in B cells, responsible for the entry of both Mg^2+^ and Ca^2+^ (Feske et al., 2012, 2015). Even though the role of Mg^2+^ has not been thoroughly studied, it is known that the lack of TRP7M in B cells induces cell death after 24 h, suggesting that the role of Mg^2+^ in B cells might be essential for cell survival (Brandao et al., 2013). The TRP7M might be essential for cell survival since the influx of Mg^2+^ induces the phosphorylation of targets from the signaling pathway involved with the tyrosine kinase-based receptors, leading to cell differentiation, growth and function (Zou et al., 2019). Interestingly, the lack of the channel TRPM7 has been associated with an increase of MAGT1 in B cells (Zou et al., 2019), a phenomenon that has not been described yet for T cells.

#### Potassium Channels

As seen for T cells, the influx of Ca^2+^ in B cells is highly dependent on the transmembrane electrostatic potential (Feske et al., 2012). K^+^ channels control the depolarization of the B cell membrane through the efflux of K^+^, and the channels involved in this process are K_v_1.3 and K_ca_3.1 (Feske et al., 2012, 2015). K_v_1.3 channels are expressed in proliferating B cells producing autoantibodies and switching to memory B cells (Wulff et al., 2004; Land et al., 2017). Blockade of this channel decreases the release of autoantibodies and has been related to inhibiting the proliferation of class-switched memory B cells (Land et al., 2017). Additionally, it has been suggested that blocking K_v_1.3 channels might help controlling multiple sclerosis, a disease in which the proliferation of class-switched memory cells is detrimental (Beeton and Chandy, 2005; Feske et al., 2012).

Importantly, although B cells express additional ion channels such as Zn^2+^ channels -i.e., ZIP3, ZIP6, ZIP8, ZIP10, ZIP14, their contribution to B cell function remains unknown (Feske et al., 2015).

#### Anionic Channels

CFTR is the only Cl^–^ channel described on the surface of B cells (Feske et al., 2012). Absence of this channel leads to the inhibition of conductance of Cl^–^, decreasing B cell activation and proliferation (Polverino et al., 2019). No other anionic channels have been described to be expressed by these cells.

## Contribution of Ion Channels to the Immunological Synapse and Other T Cell Functions

One of the most relevant processes that initiate an adaptive immune response is the interaction between antigen-presenting cells (particularly DCs) and T cells (González et al., 2007; Carreño et al., 2010, 2011). This interaction requires ions to be mobilized from several compartments and considers the participation of ion channels and transporters (Quintana et al., 2011). The contribution of ion channels and transporters (mainly focused on Ca^2+^), and the function of these channels during the immunological synapsis and other processes such as T cell activation, proliferation, and differentiation, will be discussed in this section.

### Interaction Between Dendritic Cells and T Cells

An immunological synapsis must be established to induce the activation of an adequate adaptive immune response (González et al., 2007; Franchi et al., 2009; Carreño et al., 2010, 2011). Ion channels are needed for the proper maturation of DCs, and several issues can arise when these channels are not working properly. For instance, inhibition of K_v_1.3 results in an impairment in the expression of maturation markers, MHC-II expression, and secretion of cytokines (RamaKrishnan and Sankaranarayanan, 2016). Impairment of all these characteristics will cause a reduced activation and proliferation of T cells (RamaKrishnan and Sankaranarayanan, 2016). TRPM4 is required for the migration of peripheral DCs into the lymph nodes by activating PLC (Barbet et al., 2008). Therefore, a mutation or deletion of TRPM4 in DCs impairs the assembly of the immunological synapsis in lymph nodes, because DCs fail to reach this organ (Barbet et al., 2008). Ca_v_1.2 will activate Ryanodine receptors, leading to the expression of MHC-II on the plasma membrane of DCs (Vukcevic et al., 2008). A mutation in the gene encoding for this channel would cause MHC-II not to be expressed on the DC membrane, antigen presentation would not occur, preventing the establishment of the immunological synapsis (RamaKrishnan and Sankaranarayanan, 2016).

Upon establishment of the immunological synapsis, regions known as central supramolecular activation clusters (cSMACs) are formed in the immediate vicinity of the T cell receptor-pMHC complex and concentrate most CD28 and T cell receptor molecules (Grakoui et al., 1999). Surrounding the immunological synapsis, peripheral supramolecular activation clusters emerge, where integrins such as CD2 and different intercellular adhesion molecules are located (Grakoui et al., 1999). At the most peripheric region, distant supramolecular activation clusters are formed, consisting mostly of CD45 and CD43 (Grakoui et al., 1999). One of the main indirect effects of the entry of Ca^2+^ into the T cell is the reorganization of actin on the three SMACs, modulating the intensity and the duration of T cell receptor signaling (Billadeau et al.,2007). After activation of T lymphocytes, there is a release of Ca^2+^ from the ER into the cytoplasm that leads to the activation of CRAC channel (Smith-Garvin et al., 2009). This will induce changes in the membrane potential, inducing the activation of voltage-dependent ion channels, such as K_v_1.3. This will allow the exit of K^+^ to the extracellular space and maintain the T cell membrane potential (Grissmer et al., 1993). The primary function of intracellular Ca^2+^ influx is the mobilization of the centrosome to the cSMAC (Kloc et al., 2014). The centrosome is a cellular organelle that works as the microtubule-organizing center and is spatially close to the Golgi apparatus and several vesicles (Kloc et al., 2014). The mechanism by which the centrosome is mobilized due to Ca^2+^ flux is not entirely clear. Actin interaction with myosin generates a contraction that promotes that the centrosome, along with the Golgi apparatus, move toward the cSMAC (Billadeau et al., 2007). Because the centrosome moves toward the cSMAC, the T cell can release the proper cytokines in a localized and focused way toward the antigen-presenting cells or target cell because microtubule-organizing center carries with it the Golgi and vesicles containing cytokines (Martín-Cófreces et al., 2008; Kloc et al., 2014).

The interaction between DCs and T cells is also modulated by the intracellular concentration of Mg^2+^, Zn^2+^, and Ca^2+^ and the activity of ion channels and transporters (Feske et al., 2012; Figure 2). These cations can act as second messengers modulating various T cell functions, such as cytotoxicity, differentiation, and cytokine production (Feske et al., 2012). After T cell receptor activation, MAGT1 elicits a specific influx of Mg^2+^, which activates PLCγ1 and consequently the influx of Ca^2+^ after T cell receptor activation (Feske et al., 2012). The increase in Ca^2+^ in the cytosol after T cell receptor engagement will trigger various signaling pathways leading to the T cell activation and the induction of the immune response in a T cell-dependent manner (Joseph et al., 2014). However, the mechanisms involved in activating the MAGT1 transporter are not clear yet (Feske et al., 2012). Figure 2 describes the events occurring upon T cell receptor engagement by the cognate pMHC complex, focusing on Ca^2+^ signaling.

After T cell receptor engagement, there is an influx of Ca^2+^ producing a significant increase of intracellular Ca^2+^ concentration (around 50–100 fold increase as compared to resting values; Feske et al., 2012). This phenomenon promotes the activation of transcription factors and signal transduction molecules. Among the different channels that modulate this influx are TRP channels, Ca_v_ channels, P2X channels, and CRAC channels (Feske et al., 2012). To maintain the electrical gradient necessary for the transportation of this ion, Na^+^, Cl^–^, and K^+^ channels work alongside these Ca^2+^ channels (Feske et al., 2012; Vaeth et al., 2020; Figure 2).

T cell receptor engagement leads to the depletion of Ca^2+^ stores within the ER and the phosphorylation of PLCγ (Yang and Jafri, 2020). Although two isoforms of PLCγ have been described, namely PLCγ1 and PLCγ2 (Joseph et al., 2014) T cells mainly express PLCγ1 (Joseph et al., 2014). The activation of a tyrosine kinase promotes the PLCγ1 phosphorylation, and therein, its activation (Vaeth et al., 2020; Yang and Jafri, 2020). PLCγ1 hydrolyzes phosphatidylinositol 4,5-bisphosphate (PIP_2_) into DAG and IP_3_, molecules that act as second messengers in these cells (Vaeth et al., 2020; Yang and Jafri, 2020). DAG activates the protein kinase C (PKC) and the mitogen-activated protein kinase/extracellular signal-regulated kinase (MAPK/ERK) pathways, while IP_3_ binds to IP_3_R on the ER membrane. The IP_3_R is an ion channel permeable to Ca^2+^, which causes the release of this cation from this organelle as discussed above, with the ER later being refilled by the SERCA pump to recover original Ca^2+^ levels (Joseph et al., 2014; Vaeth et al., 2020; Yang and Jafri, 2020; Figure 2).

Phospholipase Cγ is composed of three SH domains needed to achieve proper phosphorylation of the phospholipase (Joseph et al., 2014). The activation of PLCγ occurs at microclusters in T cells, which are small protein aggregates promoted by the phosphorylation of the ζ-chain of the ZAP-70 protein (Joseph et al., 2014). The events occurring before phosphorylation are related to the activation of the ZAP-70 protein due to the phosphorylation of the immune receptor tyrosine activating motif, which is mediated by the Src family kinases (Joseph et al., 2014). This family of kinases is also in charge of the phosphorylation of NMDAR subunits in hippocampal neurons (Zainullina et al., 2011). Since Src proteins are expressed in both cell types, they might also regulate the activity of NMDAR in T cells (Zainullina et al., 2011). Following antigen stimulation, these receptors regulate T lymphocyte functions and contribute to the Ca^2+^ influx mediated by the store-operated Ca^2+^ entry (Zainullina et al., 2011; Joseph et al., 2014; Fenninger and Jefferies, 2019). Significantly, the activation of PLCγ at microclusters depends on the linker for activation of T cells, the SH domain-containing leukocyte protein of 76 KDa, Vav1, guanine nucleotide exchange factor, c-Cbl, and Itk (Joseph et al., 2014). In summary, PLCγ needs to be phosphorylated to begin the mobilization of Ca^2+^ from the ER Ca^2+^ stores into the cytoplasm, increasing the intracellular Ca^2+^concentration ([Ca^2+^]_i_).

### Proliferation of T Cells

As described above, the influx of Ca^2+^ into T cells is dependent on the transmembrane electrostatic potential (Feske et al., 2012). If the transmembrane electrostatic potential is negative, Ca^2+^ may enter the cell (Feske et al., 2012; Yang and Jafri, 2020). However, the influx of Ca^2+^ promotes a transient depolarization of the membrane, which does not favor ion entry (Feske et al., 2015). K^+^ channels contribute significantly to this process because they compensate this depolarization by maintaining a negative voltage in the T cell membrane and even hyperpolarizing it through the efflux of K^+^, providing a driving force that allows Ca^2+^ entry through CRAC channels (Fanger et al., 2001; Cahalan and Chandy, 2009; Lam and Wulff, 2011; Feske et al., 2012, 2015; Teisseyre et al., 2019; Yang and Jafri, 2020). There is some controversy about the role that K^+^ channels play with regards to Ca^+2^ influx and a clamp in membrane potential caused by the opening of K^+^ channels may attenuate Ca^+2^ entry (Cahalan and Chandy, 2009). The movement of ions through membranes depends on various factors, such as the concentration gradient, the membrane potential, and the permeable ions involved (Bowman and Baglioni, 1984). All of these factors can be grouped in the electro-diffusion theory, which is the basis of the Goldman-Hodgkin-Katz equation (Bowman and Baglioni, 1984). There is not much information regarding K^+^ efflux and Ca^+2^ influx in T cells, but these two ions have been studied in non-excitable cells (HeLa cells) using a path-clamp measurement to determine membrane potential while stimulating the cell with a femtosecond laser (Ando et al., 2009). One of the main conclusions was that after laser irradiation on HeLa cells, an hyperpolarization of membrane potential correlates with a rise in intracellular Ca^+2^ (Ando et al., 2009). The crucial role that K^+^ channels play in the modulation of Ca^2+^ entry through CRAC channels allows its influx and further signaling, promoting the proliferation of T cells.

In T cells, K^+^ efflux is controlled by the channels K_v_1.3, K_*C*__a_3.1, K_2__*p*_3.1, K_2__*p*_5.1, and K_2__*p*_9.1 (Feske et al., 2015). The two most commonly expressed channels in the membrane of T cells are K_v_1.3 and K_*C*__a_3.1 (Feske et al., 2012; Conforti, 2017). Several studies have evaluated the impact of blocking these channels on T cell function and the effects vary depending on the channel that was inhibited (Petho et al., 2016). For example, the simultaneous blockage of the channels K_v_1.3, K_*C*__a_3.1, and CRAC can prevent T cell proliferation, while only blocking K_v_1.3 alters the secretion of cytokines by CD4^+^ T cells (Petho et al., 2016; Veytia-Bucheli et al., 2018). Interestingly, the activation of K_*C*__a_3.1 channels in CD8^+^ T cells modulates their chemotactic activity toward tumor cells, suggesting that the increased activity of this channel might promote CD8^+^ T cell infiltration (Chimote et al., 2018). Additionally, pharmacological inhibition of both K^+^ channels in human CD4^+^ T cells induces a decrease in IL-2 production (Valle-Reyes et al., 2018). This decrease in IL-2 correlates with the inhibition of T cell proliferation primarily in the early stages of T cell activation (Verheugen et al., 1997).

Naïve T cells express mainly K_v_1.3 channels, and upon T cell receptor engagement, there is an upregulation and recruitment of K_Ca_3.1 channel into the IS (Feske et al., 2015). Membrane depolarization by the influx of Ca^2+^ is sensed by a set of arginine residues in the transmembrane segment four of these channels, causing conformational changes and their opening. Interestingly, the activation of this channel is also regulated by Lck, PKC ζ, and PKA (Nicolaou et al., 2010; Feske et al., 2012, 2015).

The binding of Ca^2+^ and calmodulin inside T cells activates the K_Ca_ 3.1 channel, as the C terminus of this channel is constitutively linked to calmodulin (Feske et al., 2012). The activation of this channel is also dependent on PKA and the class 2 phosphatidylinositol-3-kinase (PI3K-C2β), which is activated after T cell receptor stimulation (Feske et al., 2012). The activation of PI3K-C2β increases PI_3_P levels in the plasma membrane, allowing the histidine kinase nucleoside diphosphate kinase B to activate the K_Ca_ 3.1 channel by phosphorylating histidine 358 in its carboxyl terminus (Feske et al., 2012, 2015). Accordingly, dephosphorylation of PI_3_P and K_Ca_ 3.1 inhibits the activity of this channel, T cell proliferation, and Ca^2+^ influx induced by T cell receptor signaling (Feske et al., 2012, 2015). Such an inhibition occurs through the PI_3_P phosphatase myotubularin-related protein 6 and the histidine phosphatase phosphohistidine phosphatase-1. K_Ca_ 3.1 channels can also be inhibited through the ubiquitination and inhibition of PI3K-C2β due to the E3 ubiquitin ligase tripartite motif-containing protein 27 (Feske et al., 2012, 2015).

### T Cell Polarization

The overall immune response must be tightly regulated to recognize and clear different pathogens. CD4^+^ T cells are usually in charge of orchestrating this response by polarizing into different Th profiles, with key cytokines secreted by each of these profiles (Gálvez et al., 2021). For instance, while a Th1 polarization induces the secretion of IFN-γ and will be optimal for the clearance of intracellular pathogens, a Th2 response induces the secretion of IL-4 and will be optimal for the clearance of extracellular bacteria and a Th17 response induces the secretion of IL-17 and will be optimal for the clearance of extracellular pathogens, such as parasites (Gálvez et al., 2021). Interestingly, the absence of TRPA1 induces the polarization of T cells into a Th1 profile, increasing the expression of T-bet and IFN-γ (Bertin et al., 2017). The role of ion channels modulating T cell profiles toward a Th2 response has been associated with the upregulation of Ca_v_ channels mentioned above (Feske et al., 2012). The role of some ion channels during Th17 polarization has been partially described up to date. The entry of Ca^2+^ through the P2 × 7 receptor activates ERK1 or ERK2, which suppresses the transcription of forkhead box P3 and promotes the expression of retinoic acid receptor-related orphan receptor-γt, which induces the polarization of T cells toward a Th17 phenotype, altogether inhibiting the regulatory T cells (T_reg_) polarization (Feske et al., 2012).

### Cytokine Secretion by T Cells

Translocation of different transcription factors, such as the nuclear factor of activated T cells (NFAT) and the nuclear factor kappa-light-chain-enhancer of activated B cells (NF-κB) into the nucleus will induce the transcription of genes encoding for cytokines, such as IL-2 (Vaeth et al., 2020; Yang and Jafri, 2020). Ca^2+^ influx through CRAC channels regulates the serine/threonine phosphatase calcineurin function, which coordinates three main signaling pathways (Vaeth et al., 2020; Yang and Jafri, 2020). The first pathway is related to NFAT signaling, the second one is associated with NF-κB signaling and the third one is related to the c-Jun NH_2_-terminal kinase (JNK) pathway (Vaeth et al., 2020; Yang and Jafri, 2020). For the first pathway, calcineurin dephosphorylates NFAT, specifically the regulatory domain in the N terminal (Hogan et al., 2003). This exposes nuclear localization signals, promoting the translocation of this complex toward the nucleus, eventually inducing the transcription of cytokine genes, such as IL-2 (Joseph et al., 2014; Yang and Jafri, 2020). To activate this particular transcription pathway, T cells need a low and extended intracellular Ca^2+^ influx (Joseph et al., 2014; Yang and Jafri, 2020). Certain distal regulatory elements in NFAT target genes from T cells (mainly enhancers) can be found in cytokine genes, including IL-3, GM-CSF, IL-4, IL-10, and IFN-γ genes (Hogan et al., 2003). It is also important to indicate that NFAT can interact with other transcription factors, such as the activator protein 1 (AP-1) transcription factor, ICER, EGR, and GATA (Hogan et al., 2003). Calcineurin also regulates NF-κB signaling pathway, as an increase in intracellular Ca^2+^ promotes PKCα, which along with protein kinase C theta (PKCθ; previously activated by the co-stimulation of the T cell receptor/CD3 and CD28) co-activates the Iκ kinase (IKK) and phosphorylates IκB (Yang and Jafri, 2020). Phosphorylation induces IκB dissociation from NF-κB, leading to IκB polyubiquitination and degradation by the proteasome (Yang and Jafri, 2020). Therefore, NF-κB is initially retained in the cytoplasm because of the association with the inhibitory IκB protein, but later on, after the dissociation from IκB, free NF-κB translocates into the nucleus and promotes gene transcription (Yang and Jafri, 2020). This mechanism for the activation of gene transcription is called the canonical NF-κB activation pathway (Brasier, 2006). To activate this transcription factor, unlike for NFAT signaling pathway, T cells need a high and short increase in Ca^2+^ ions in the cytoplasm (Joseph et al., 2014; Yang and Jafri, 2020; Figure 2). Using Hodgkin’s cell lines, seventeen novel genes regulated by NF-κB were described, such as IL-13, MDC, I-309, EMR, CD44, and the transcription factors STAT5_a_, IRF-1, Spi-B, and LITAF (Brasier, 2006).

Another pathway related to Ca^2+^ influx is the JNK pathway. Activation of c-Jun modulated by PKCθ regulates MAPK cascade of SEK1/MKK4 and phosphorylates JNK (Yang and Jafri, 2020). This will trigger JNK signaling, along with calcineurin, which also activates SEK (Yang and Jafri, 2020). SEK phosphorylates two serine residues in the activation domain of c-Jun, promoting the activation of this protein (Yang and Jafri, 2020). c-Jun, along with c-Fos establishes the AP-1 transcription factor complex, which plays a role in cell growth and IL-2 induction (Avraham et al., 1998; Yang and Jafri, 2020). The activation of JNK can be detected minutes after pMHC- T cell receptor interaction, along with the binding of B7-1 and -2 molecules to the CD28 receptor on T cells. Both signals are needed to strongly activate JNK as they synergize (Avraham et al., 1998).

All this coordinated activation of NFAT, NF-κB, and AP-1 will result in the activation of pathways that will induce the secretion of several cytokines, including IL-2, IL-8, IL-1β, IL-4, IL-6, IFN-γ, and TNF-α (Fiebich et al., 2012). Among all of these cytokines, the secretion of IL-2 is essential because it modulates the differentiation of CD4^+^ T cells toward a Th2 or Th1 profile while inhibiting Th17 differentiation (Fiebich et al., 2012).

## Diseases Associated to Deficiencies in Ion Channels and Transporters

Diseases associated with ion channels and transporters mutations that change the molecular structure, topology, and functions of these proteins are called channelopathies (Kim, 2014; Schorge, 2018; Vaeth and Feske, 2018). These diseases are commonly caused by mutations in the alpha subunits or the accessory proteins of these channels (Kim, 2014; Schorge, 2018; Vaeth and Feske, 2018). Channelopathies have been linked to CNS diseases (i.e., epilepsy, ataxia, and migraine), heart diseases, lung diseases (i.e., cystic fibrosis), liver disease, and kidney diseases (Kim, 2014; Schorge, 2018; Vaeth and Feske, 2018). Channelopathies can also affect the immune system, causing immune alterations, such as autoimmune diseases or immunodeficiencies (Feske et al., 2015; Vaeth and Feske, 2018). For instance, mutations that abolish the functionality of CRAC channels, by loss of function of the ORAI1 gene or mutations in the STIM1 gene (components of this channel that we will further describe below), are responsible for a syndrome called CRAC channelopathy (Feske, 2010; Lacruz and Feske, 2015). This syndrome will lead to the establishment of combined immunodeficiency (CID), a disease that is directly linked to the absence of store-operated Ca^2+^ entry (Feske, 2010; Lacruz and Feske, 2015). CID patients, unlike severe combined immunodeficiency patients, exhibit normal counts of most immune cells, including T and B cells, and most myeloid populations, while displaying odd numbers of unconventional lymphocytic populations, such as Tregs, iNKT cells, and γδ T cells (Feske, 2010; Lacruz and Feske, 2015). Although low in number, these cell types are fundamental for establishing a proper immune response (La Cava, 2009; Nielsen et al., 2017; Gálvez et al., 2021). During their first years of life, CID infants require transplantation of hematopoietic stem cells to face the different severe diseases they develop due to their lack of these unconventional lymphocytes (Feske, 2010; Lacruz and Feske, 2015; Vaeth and Feske, 2018). These severe diseases will usually be caused by common and less pathogenic microorganisms like *Candida albicans*, *Streptococcus pneumoniae*, and cytomegalovirus (Feske, 2010; Lacruz and Feske, 2015; Vaeth and Feske, 2018). The lack of Tregs can also lead to the development of autoimmune hemolytic anemia, which originated from the presence of autoantibodies (Lian et al., 2018). Other diseases related to mutations in ion channels and transporters have been described, such as X-linked immunodeficiency with magnesium defects, Epstein-Barr virus infections, and neoplastic syndrome, which arise from the loss of function MAGT1 (Li et al., 2011; Ravell et al., 2014). Several other disorders have been described, such as agammaglobulinemia resulting from loss of function of LRRC8A (a Cl^–^ channel) (Sawada et al., 2003) and transient neonatal zinc deficiency, which significantly impairs the activation of T cells and is originated by loss of function of ZIP4 (a Zn^2+^ channel) (Dufner-Beattie et al., 2005; Geiser et al., 2012). Additionally, channelopathies not only promote the infection, but it has also been described that viral infections can lead to the alteration of cation channels, such as Ca^2+^, K^+^, and Na^+^ (Charlton et al., 2020), creating dysregulation of the concentrations of Ca^2+^ along with other concentrations of cations. Furthermore, the immune response elicited against the infection can induce channelopathies, due to the binding of autoantibodies to IC (Vaeth and Feske, 2018). The proper function of these channels is fundamental for the immune system and the overall organism.

## Modulation of Ca^2+^ During Viral Infections

As described above, Ca^2+^ is essential for numerous functions of cells. Interestingly, viruses can take advantage of this by modulating the intracellular concentration of Ca^2+^ ([Ca^2+^]_i_) used in processes such as viral replication (Zhou et al., 2009). During viral infections, there are changes on [Ca^2+^]_i_ that favor viral replication or the establishment of persistent infections on target cells, as discussed below (Zhou et al., 2009). In this section, we will describe the mechanisms used by RNA viruses, such as the human immunodeficiency virus (HIV), respiratory syncytial virus (hRSV), severe acute respiratory coronavirus 2 (SARS-CoV2), and DNA viruses, such as hepatitis B virus (HBV), and herpes simplex virus (HSV) to modulate the concentration of Ca^2+^ altering the cellular signaling.

### RNA Viruses

#### Human Immunodeficiency Virus

**FIGURE 3:**
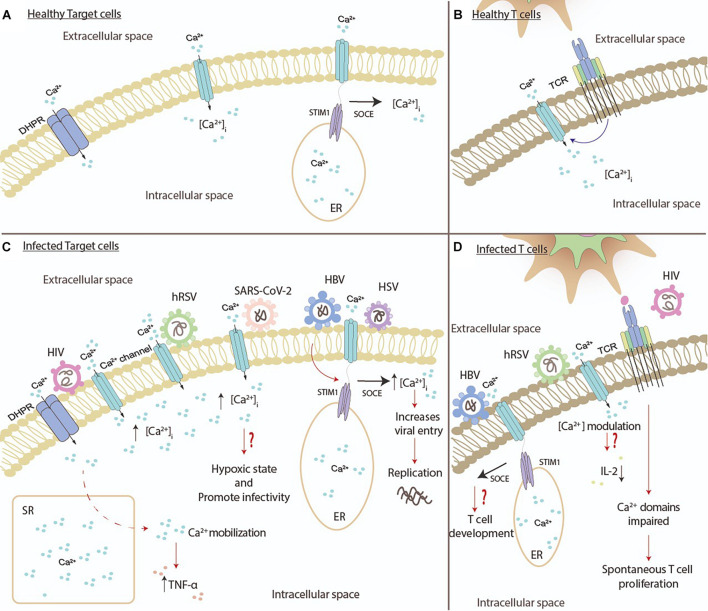
Viral pathogens modify biological processes on immune cells by increasing the Ca^2+^. **(A)** During normal conditions, various immune cells, such as dendritic cells, monocytes, macrophages, and neutrophils, regulate their concentrations of intracellular Ca^2+^. The activation of dihydropyridine receptor (DHPR) and Ca^2+^ channels promote the entry of Ca^2+^ into the cells. The interaction between the CRAC channel and STIM1 promotes the movement of Ca^2+^ into the cytosol. **(B)** During normal conditions, T cells regulate their concentrations of intracellular Ca^2+^ through the activation of the T cell receptor (TCR). **(C)** Different viruses can modulate different processes in various immune cells. The human immunodeficiency virus (HIV) can increase the Ca2+ concentration through the modulation of Ca^2+^ channels, and the activation of DHPR promotes the mobilization of Ca^2+^ that leads to the secretion of TNF-α. The human respiratory syncytial virus (hRSV) promotes the increase of Ca^2+^ as well. SARS-CoV-2 might be causing a hypoxic state and increase the infectivity of the virus through the increase Ca^2+^ concentration. The hepatitis B virus (HBV) and herpes simplex virus (HSV) can increase the concentration of intracellular Ca^2+^ through the interaction between the CRAC channel and STIM1. **(D)** RNA viruses can modulate different processes in T cells. HIV proteins activate the T cell receptor (TCR), and as a result, it impairs the domains of Ca^2+^, leading to spontaneous T cell proliferation. hRSV induces the influx of Ca^2+^ into T cells, which might decrease the secretion of IL-2. As for DNA viruses, such as HBV, the interaction between the CRAC channel and STIM1 might modulate the development of T cells.

Human immunodeficiency virus is a relevant virus transmitted through sexual intercourse, blood perfusion, needle sharing, and vertical transmission. This virus belongs to the *Retroviridae* family, and its genome is composed of two positive-sense single-stranded RNA molecules encoding for three polyproteins (Gag, Pol, and Env) and six accessory proteins (Tat, Rev, Nef, Vpr, Vif, and Vpu; Frankel and Young, 1998; German Advisory Committee Blood (Arbeitskreis Blut), 2016; Rossi et al., 2021). The main target of infection of HIV are CD4^+^ cells and CCR5- or CXCR4-positive cells, including DCs, macrophages, T helper cells, monocytes, microglia, and astrocytes (German Advisory Committee Blood (Arbeitskreis Blut), 2016; Rossi et al., 2021). Like other viruses, HIV modulates [Ca^2+^] and Ca^2+^-dependent channels during different stages of infection, including replication, assembly, budding, and also during immune response evasion (German Advisory Committee Blood (Arbeitskreis Blut), 2016; Chen et al., 2019). It has been described that HIV proteins have a role in Ca^2+^ modulation. Both HIV proteins gp120 and Tat can increase [Ca^2+^]_i_ by modulating Ca^2+^ channels in several cell types, such as immune cells, astrocytes, neurons, and epithelial cells (Zocchi et al., 1998; Contreras et al., 2005; Zhou et al., 2009; Hu, 2016; Cai et al., 2020). In normal conditions, DCs need the Ca^2+^ mobilization for several function such as the engulfment of apoptotic bodies and antigen presentation (Poggi et al., 1998). Accordingly, the voltage-sensitive *dihydropyridine* receptor (DHPR) L-type calcium channel expressed in human monocyte-derived DCs (mo-DCs) plays an essential role in Ca^2+^ mobilization. This function was demonstrated by the use of nifedipine (NFP) which inhibit the DPHR function, affecting Ca^2+^ mobilization and engulfment of apoptotic bodies by mo-DCs (Figure 3; Poggi et al., 1998). The same phenomenon was observed when mo-DCs were stimulated with HIV Tat protein, which suggests that this protein can interact with DHPR, prevent the entry of Ca^2+^ into the cells, and affect essential functions, such as apoptotic body engulfment and IL-12 secretion (Poggi et al., 1998).

Moreover, human NK cells express a functional phenylethylamine-sensitive (PAA) L-type calcium channel, which when blocked with verapamil (VPM; L-type calcium channel antagonist) the Ca^2+^ influx from the extracellular media, the tumor cell killing function was affected (Zocchi et al., 1998). The interaction between human NK cells and HIV Tat protein induces a decrease of Ca^2+^ influx, promoting the inhibition of Ca^2+^-dependent functions, such as NK cell cytotoxicity, as seen with VPM (Zocchi et al., 1998). Like other immune cells, human monocytes stimulated with Tat can mobilize Ca^2+^ from the extracellular media (Contreras et al., 2005). Additionally, human monocytes and other immune cells express a functional DHPR in the cellular membrane surface, which has an essential role in cytokine secretion (Contreras et al., 2005). HIV Tat protein uses DHPR to induce the Ca^2+^ mobilization, promoting the induction of TNF-α production by human monocytes during infections (Figure 3; Contreras et al., 2005).

On the other hand, because HIV can induce neurotoxicity, using a recombinant HIV gp120 protein in primary human cultures of astrocytes and neurons has been shown to increase [Ca^2+^]_i_, which occurs first in astrocytes and then in neurons, and also that NMDARs may mediate this effect in both cells types (Holden et al., 1999). Only in neurons there exists participation of the L-type calcium channel, as astrocytes do not express it. These results suggest that gp120 can modulate [Ca^2+^] influx through Ca^2+^ channels and NMDARs, promoting neurotoxicity (Holden et al., 1999).

Another important HIV protein is Nef, which induces T cell activation, which is necessary for viral replication in these cells and promotes hyperactivation in the absence of CD28 ligation (Manninen and Saksela, 2002; Fenard et al., 2005). In Jurkat cells, HIV Nef can induce an increase of Ca^2+^, mediated by the interaction with IP_3_R (Manninen and Saksela, 2002; Zhou et al., 2009). Furthermore, it has been described that Nef is found in lipid rafts, which are recruited into the IS space minutes after T cell receptor engagement (Figure 3; Fenard et al., 2005). Besides, HIV Nef can impair the formation of Ca^2+^ domains after T cell receptor engagement, suggesting that this viral protein can control the cellular distribution of Ca^2+^ levels, allowing hyperactivation, which implies a spontaneous T cells proliferation, including the expression of the T cells activation antigens and increased cytokines secretion, among other changes (Figure 3; Ott et al., 1997; Fenard et al., 2005).

#### Human Respiratory Syncytial Virus

One of the most prevalent viral agents that causes acute lower tract respiratory infections in infants under 2 years old, as wells as immunocompromised and elderly individuals, is hRSV (Tabor et al., 2021). Human RSV is an enveloped, negative-sense single-strand RNA virus that belongs to the *Pneumoviridae* family. This virus encodes for eleven proteins, including three in the virion surface (F, G, and SH) and four proteins found inside the viral structure (N, L, P, and M2.1; Collins et al., 2013; Cui et al., 2016). Decades ago, it was described that syncytia formation, characteristic of hRSV infection, in HEp-2 cells requires the presence of Ca^2+^ (Shahrabadi and Lee, 1988). Later, using a genetically-encoded Ca^2+^ indicator strategy in hRSV-infected MA104 cells, observing increases of [Ca^2+^]_i_ and Ca^2+^ influx during cell fusions (Figure 3; Perry et al., 2015). Ca^2+^ concentrations increased first in the cytoplasm and then in the ER, and a rapid increase of Ca^2+^ influx before cell death was observed (Perry et al., 2015). Accordingly, the results suggest that hRSV may modulate Ca^2+^ levels. Additionally, hRSV infection requires the host Ca^2+^ pump SPCA1 located in the *trans*-Golgi network. A deficiency of SPCA1 has a relevant impact on hRSV spread and alters the susceptibility of the cells to infection, corroborating the crucial role of Ca^2+^ (Hoffmann et al., 2017). Additionally, hRSV infection of human epithelial cells, such as BEAS-2B, has been shown to increase upon expression of TRPV1, which is implicated in asthma predisposition (Omar et al., 2017; Harford et al., 2018). With regards to the ability of hRSV to modulate ions, it is known that the SH protein is a viroporin that forms pentameric cation-selective ion channel at the surface of the infected cells, allowing the entry of Na^+^ and K^+^ and promoting the activation of the NLRP3 inflammasome (Gan et al., 2012; Triantafilou et al., 2013).

As described above, the modulation of [Ca^2+^]_i_ through the expression or the function of ion channel and pumps by hRSV can affect the immune response. It is known that hRSV can impair immunological synapsis by recruiting the N protein to the surface of the infected DCs (Gonzalez et al., 2008; Céspedes et al., 2014). Moreover, recent evidence shows that hRSV can infect human T cells (CD4^+^ and CD8^+^) and decrease IL-2 production, which may explain the absence of a long-lasting protective immunity (Figure 3; Raiden et al., 2017). Regarding the relevance of Ca^2+^ for hRSV infection, currently, there are no studies evaluating whether hRSV infection can modulate [Ca^2+^]_i_ or Ca^2+^ signaling pathways in infected DCs or T cells.

#### Severe Acute Respiratory Coronavirus 2

The newly described coronavirus SARS-CoV-2 is the causative agent of an unprecedented pandemic in the last 100 years. This virus is an enveloped particle with a single-strand positive-sense RNA that encodes four structural proteins (S, E, M, and N) and sixteen non-structural proteins (nsp1-16; McClenaghan et al., 2020; Wang et al., 2020). Patients admitted to hospitals show low serum Ca^2+^, which is currently a severity indicator (Zhou et al., 2020). Moreover, it has been described that SARS-CoV-2 infection induces hypoxia in epithelial cells and innate immune cells (Serebrovska et al., 2020; Danta, 2021), and in a hypoxic state, there is an increase in [Ca^2+^]_i_, mediated by CRAC channels (Figure 3; Gusarova et al., 2011; Danta, 2021). Previously, it was described that SARS-CoV and MERS- CoV could alter Ca^2+^ concentration, which induced the viral entry into target cells, promoting viral fusion and, therein, increase their infectivity (Millet and Whittaker, 2018; Straus et al., 2020b). Furthermore, it was reported that the E protein of SARS-CoV is a viroporin that, like the hRSV SH protein, forms a pentameric ion channel that shows higher selectivity for Ca^2+^ than Na^+^ and K^+^ (Nieto-Torres et al., 2015). Indeed, the IC formed by the E protein can transport the Ca^2+^, which allows the subsequent activation of the NLRP3 inflammasome (Nieto-Torres et al., 2015). The SARS-CoV2-E (2-E) protein also forms an ion channel permeable to cations such as K^+^, Na^+^, and Ca^2+^ and causes cell death (Xia et al., 2020). Moreover, the 2-E protein by itself can induce the secretion of pro-inflammatory cytokines by macrophages (Xia et al., 2020).

The relevance of the Ca^2+^ channel for SARS-CoV-2 infection has been observed in Vero E6 and Calu-3 cells, and the use of calcium channels blockers, such as amlodipine, felodipine, and nifedipine, has been studied (Straus et al., 2020a). These Ca^2+^ channels blockers act by inhibiting the L-type Ca^2+^ channel, modulating viral entry into the cells, and viral replication, thus being a promising and potential therapeutic alternative for SARS-CoV-2 infection (Straus et al., 2020a). Although the main cellular targets for SARS-CoV-2 infection are epithelial cells, immune cells such as neutrophils, monocytes, and T cells are also susceptible to infection (Borsa and Mazet, 2020; Pontelli et al., 2020). How SARS-CoV-2 can modulate Ca^2+^ flux or interact with ion channels in infected immune cells remains to be elucidated.

### DNA Viruses

#### Hepatitis B Virus

Hepatitis B virus infection is a common cause of hepatic pathologies, such as hepatitis, cirrhosis, and hepatocellular cancer, which are public health problems worldwide (Hou et al., 2005; Liang, 2009). This virus is transmitted by contact with infected body fluids, such as blood (Hou et al., 2005). HBV is a small DNA enveloped virus with an icosahedral nucleocapsid that belongs to the *Hepadnaviradae* family (Venkatakrishnan and Zlotnick, 2016). This virus encodes for three envelop proteins (L, M, and S) and for the preCore, core, pol, and X (HBx; Seeger and Mason, 2015). Like other viruses, HBV modulates Ca^2+^ concentration to promote viral replication in hepatocytes. Remarkably, the HBx protein has been implicated in Ca^2+^ modulation and signaling (Bouchard et al., 2001; Casciano et al., 2017). In this context, it has been reported that HBx exerts effects on intracellular stored Ca^2+^, where it induces signal transduction pathways to allow HBV replication (Bouchard et al., 2001). Accordingly, HBx, not only by itself but in an HBV replication context, can interact with mitochondria and probably through the modulation of the mitochondrial permeability transition pore, control [Ca^2+^]_i_ influx (McClain et al., 2007). Data obtained from rat primary hepatocytes showed that HBx could increase [Ca^2+^]_i_ altering IP_3_-linked Ca^2+^ responses and modulate Ca^2+^ influx through the store-operated Ca^2+^ channels, which is necessary for HBV replication (Figure 3; Casciano et al., 2017). Furthermore, in HEK 293 cells, the interaction between HBx and ORAI1 is essential to maintain active the store-operated Ca^2+^ entry (Yao et al., 2018). According to this, the co-localization of HBx with ORAI1 suggests an upregulation of the STIM1-ORAI1 complex that modulates store-operated Ca^2+^channels positively, especially when Ca^2+^ concentrations are low, to induce an intracellular influx (Figure 3; Yao et al., 2018).

Hepatitis B virus can infect and replicate in PBMCs, including T cells, affecting the immune response against this virus (Yan et al., 2016). As described early, for the development and function of T cells and DCs, the role of store-operated Ca^2+^ entry is crucial for establishing the immunological synapsis (Figure 3; Oh-Hora, 2009; Félix et al., 2013). However, there are no studies that evaluate the role of HBx in immune cells.

#### Herpes Simplex Viruses

One of the most ubiquitous human infections in the mucocutaneous tissue is caused by herpes simplex viruses type 1 and type 2 (HSV-1 and HSV-2) (He et al., 2020). HSVs belong to the *Herpesviradae* family and have a linear double-stranded DNA genome packaged in an icosahedral capsid that is enveloped. The envelope has numerous proteins, and the virus encodes twelve different glycoproteins (gB, gC, gD, gE, gG, gH, gI, gJ, gK, gL, gM, and gN; Karasneh and Shukla, 2011; Retamal-Díaz et al., 2016; Madavaraju et al., 2021). HSVs can infect several cell types, including epithelial cells, fibroblasts, neurons, and DCs (Koelle et al., 1993; Karasneh and Shukla, 2011; Retamal-Díaz et al., 2016; Marcocci et al., 2020).

Herpes simplex viruses infection of epithelial cell lines induces a transient increase in [Ca^2+^]_i_, mediated by the ER and Ca^2+^ influx from extracellular space (Figure 3; Cheshenko et al., 2003). Additionally, IP_3_-sensitive Ca^2+^ stores, probably available through the opening of Ca^2+^ channels in the ER membrane, help HSV enter the cell. On the other hand, the chelation of Ca^2+^ and pharmacological inhibition of IP_3_R impairs viral infection (Cheshenko et al., 2003). ND7/23 cells (sensory-like neurons) express the Ca_v_3.2 T-type Ca^2+^ channel that regulates the excitability of neurons. Significantly, the expression of this channel is reduced by HSV-1 infection, which depends on viral replication and protein synthesis (Zhang et al., 2019). HSV-2 infection in HeLa cells promotes an extracellular Ca^2+^ influx, which can be blocked by the use of Ca^2+^ channels blockers and, therein, suppresses viral infection. T-type, but not L-type Ca^2+^ channel, impact HSV-2 infection, as proven by the use of Ca^2+^ channels blockers (Ding et al., 2021). Regarding the effects of HSV-1 on immune cells, it has been reported that these cells express the herpesvirus entry mediator, which is a receptor for the HSV-1 gD, allowing viral entry into DCs and activated T cells (La et al., 2002). Besides, it is known that HSV infection of DCs can modulate the maturation and their capacity of activating T cells, and also cause DC death (Jones et al., 2003; Bosnjak et al., 2005; Retamal-Díaz et al., 2016), but there is no data to date regarding Ca^2+^ modulation and the role of Ca^2+^ channels involved in this process. On the other hand, T cell proliferation can be modulated by the HSV-1 gD protein (Bosnjak et al., 2005). Additionally, HSV infection of T cells, in which gB, gD, gH, and gL are necessary for viral entry, or contact with HSV-infected cells, such as fibroblasts, can impair T cell receptor-mediated activation by altering Ca^2+^ mobilization that is required for the T cell receptor signaling (Koelle et al., 1993; Sloan et al., 2006; Cao et al., 2008). The effects of HSV infection over Ca^2+^ channels in immune cells remains poorly understood and requires more studies to explore the potential development of new therapeutic strategies that target these components.

## Conclusion

In all cell types, ion balance is crucial to maintain normal physiology and functions. In this context, ion channels play critical roles in controlling ion homeostasis. One of the most relevant ions is Ca^2+^, which acts as a second messenger, and is crucial for eliciting a proper immune response against pathogens. Immune cells, such as T cells, express a wide variety of ions channels on their surface, including four types of Ca^2+^ channels CRAC, TRP, Ca_v_, and P2X (Feske, 2013; Feske et al., 2015). These channels are implicated in the proliferation, activation, and cytotoxicity of T cells, all crucial mechanisms for the adaptive immune response. It has been described that loss-of-function mutations in Ca^2+^ channel genes affect their function on immune cells, provoking combined immunodeficiency and increasing the susceptibility to infections (Vaeth and Feske, 2018). Importantly, viruses also need Ca^2+^ mobilization for entry, replication, and budding (Zhou et al., 2009).

Moreover, viruses such as HIV, hRSV, SARS-CoV2, HBV, and HSV, among others, have proteins that can interfere with the normal function of Ca^2+^ channels to increase [Ca^2+^]_i_ and enhance their infectivity in target cells (Zhou et al., 2009). Viral modulation of Ca^2+^ channels expressed in immune cells allows viruses to impair the immune responses as an evasion mechanism (Fenard et al., 2005; Sloan et al., 2006). Despite this knowledge, further insights into the associated mechanism, the impact of using Ca^2+^ channels blockers, and their effects on viral infections are still required.

## Author Contributions

All authors listed have made a substantial, direct and intellectual contribution to the work, and approved it for publication.

## Conflict of Interest

The authors declare that the research was conducted in the absence of any commercial or financial relationships that could be construed as a potential conflict of interest.

## Publisher’s Note

All claims expressed in this article are solely those of the authors and do not necessarily represent those of their affiliated organizations, or those of the publisher, the editors and the reviewers. Any product that may be evaluated in this article, or claim that may be made by its manufacturer, is not guaranteed or endorsed by the publisher.

## Abbreviations

Ca^2+^: Calcium
Mg^2+^: Magnesium
Zn^2+^: Zinc
K^+^: Potassium
Na^+^: Sodium
Cl^–^: Chloride
HPO4^2–^: Phosphate
HCO3^–^: Bicarbonate
CRAC: Ca^2+^ release-activated Ca^2+^ channels
TRP: Transient receptor potential
CFTR: Cystic fibrosis transmembrane conductance regulator
GABA: Gamma-aminobutyric acid
PLC β: phospholipase C β
PIP_2_: Phosphatidylinositol 4,5-bisphosphate
IP_3_: inositol (1,4,5) trisphosphate
DAG: Diacylglycerol
IP_3_R: IP_3_ receptor
ER: Endoplasmic reticulum
MAGT1: Magnesium transporter protein 1
DCs: Dendritic cells
STIM1: Stromal interaction molecule 1
pMHC: Peptide-MHC
PMCAs: Plasma membrane Ca^2+^ ATPase pumps
SERCA: Sarco-endoplasmic reticulum Ca^2+^ ATPase
ADP: Adenosine diphosphate
cADPR: cyclic ADP ribose
Panx1: Pannexin 1 hemichannels
TRPM2: TRP cation channel subfamily M member 2
NMDARs: *N*-methyl -D-aspartate receptors
AMPARs: α-amino -3-hydroxy-5-methyl-4-isoxazole propionic acid receptors
cSMACs: Central supramolecular activation clusters
PKC: Protein kinase C
PI3K-C2 β: Phosphatidylinositol-3-kinase
NFAT: Nuclear factor of activated T cells
NF- κ B: Nuclear factor kappa-light-chain-enhancer of activated B cells
JNK: c-Jun NH_2_-terminal kinase
AP-1: Activator protein 1
PKC θ: Protein kinase C theta
IKK: I κ kinase
CNS: Central nervous system
CID: Combined immunodeficiency
HIV: Human immunodeficiency virus
hRSV: Human respiratory syncytial virus
SARS-CoV2: Severe acute respiratory coronavirus 2
HBV: Hepatitis B virus
HSV: Herpes simplex virus
DHPR: Voltage-sensitive *dihydropyridine* receptor
VPM: Verapamil.
